# Public health perspectives on green efficiency through smart cities, artificial intelligence for healthcare and low carbon building materials

**DOI:** 10.3389/fpubh.2024.1440049

**Published:** 2024-12-16

**Authors:** Jingjing Sun, Xin Guan, Siqi Yuan, Yalin Guo, Yepei Tan, Yajuan Gao

**Affiliations:** ^1^School of Public Administration, Guangzhou University, Guangzhou, China; ^2^Guangzhou Xinhua University, Dongguan, China; ^3^School of Arts and Communication, Beijing Normal University, Beijing, China; ^4^School of Marxism, Wuhan University of Science and Technology, Wuhan, China; ^5^Cyberspace Institute of Advanced Technology, Guangzhou University, Guangzhou, China; ^6^School of Fine Art and Design, Guangzhou University, Guangzhou, China

**Keywords:** green benefits, smart city, artificial intelligence medical care, low-carbon building materials, convolutional neural network

## Abstract

**Introduction:**

Smart cities, artificial intelligence (AI) in healthcare, and low-carbon building materials are pivotal to public health, environmental sustainability, and green efficiency. Despite their critical importance, understanding public perceptions and attitudes toward these domains remains underexplored. Additionally, the effective use of advanced technologies like convolutional neural networks (CNN) in predicting and promoting low-carbon solutions in construction is gaining attention.

**Methods:**

This study employs a dual approach: (1) A survey of 200 respondents was conducted to gauge public perceptions and attitudes toward smart cities, AI in medicine, and low-carbon building materials. (2) A CNN model was developed and implemented to predict the performance of low-carbon building materials. The model utilized convolutional and pooling layers to capture local features and spatial information from image datasets, with tasks including image classification and segmentation.

**Results:**

The survey results indicate high awareness of smart cities (80%), with 60% associating them with environmental protection and green living. For AI in medicine, 70% of respondents are aware of its applications, but only 45% perceive it as environmentally beneficial. Regarding low-carbon building materials, 60% expressed willingness to pay premium prices, and 65% recognized their positive environmental impact. The CNN model demonstrated high prediction accuracy on both training and validation datasets, effectively aiding in the identification of low-carbon materials and reducing building energy consumption and carbon emissions.

**Discussion:**

The findings highlight significant public awareness and diverse attitudes toward these critical domains, suggesting the need for improved communication and advocacy for AI’s environmental benefits. The application of CNN models in the construction industry showcases a promising pathway to enhance material selection efficiency and foster sustainable practices. These insights are essential for aligning public understanding with technological advancements to achieve environmental and public health goals.

## 1 Introduction

As science and technology march forward, the concept of smart cities has emerged as a beacon of innovative urban development. Through the strategic utilization of information technology and data analysis, smart cities have transformed into bustling hubs of efficiency, convenience, and comfort, ultimately enhancing the quality of life for residents (1–3). Within the framework of smart city initiatives, the integration of artificial intelligence (AI) technology and the promotion of eco-friendly building materials emerge as pivotal strategies in fostering urban sustainability and environmental stewardship. As the pace of urbanization accelerates, cities grapple with a myriad of challenges ranging from traffic congestion to environmental degradation, necessitating proactive solutions. Enter smart cities, poised to confront these challenges head-on by leveraging cutting-edge technologies such as AI, communication networks, and big data analytics to intelligently manage and optimize various urban facets, including infrastructure, services, and resource allocation. This holistic approach not only presents innovative solutions but also charts a promising path toward mitigating pressing urban concerns, paving the way for a greener, more sustainable future (4–6).

As AI technology rapidly evolves, its integration into the medical field has matured significantly. AI now plays a pivotal role in aiding physicians across various aspects of medical care, from disease diagnosis to treatment planning and prognostication, thanks to its ability to analyze vast amounts of data and deploy sophisticated machine learning algorithms (7, 8). Particularly in medical imaging, AI has achieved diagnostic accuracy comparable to seasoned medical professionals and, in some cases, even surpasses human performance. Moreover, AI facilitates personalized medical care by enabling intelligent medical devices and health management systems, enhancing both the quality and efficiency of medical services for patients. This synergy between AI and medicine heralds a new era of precision medical care, where technology empowers clinicians to deliver more accurate diagnoses and tailored treatment plans, ultimately improving patient outcomes and revolutionizing the practice of medicine (9). The extensive application of AI in the medical field, such as in diagnostics and treatment planning, has indeed enhanced efficiency and accuracy. However, it may also lead to a series of issues, including but not limited to data privacy breaches, misdiagnoses due to algorithmic bias, and the potential replacement of human physicians, which could exacerbate social inequality (10, 11). Particularly, if access to and utilization of AI technologies are uneven, it may intensify the unequal distribution of healthcare resources, thereby affecting the accessibility and equity of public health services. While AI technologies have brought numerous conveniences and efficiency improvements in the short term, their long-term impacts on social structures and the environment cannot be overlooked. Specifically, the rapid development and widespread application of AI technologies may accelerate resource consumption, potentially causing irreversible harm to the environment in the absence of appropriate green technologies and policy guidance.

The greening of AI represents a direction worthy of in-depth exploration. By optimizing algorithm designs, utilizing renewable energy-poared data centers, and developing more environmentally friendly hardware materials, it is possible to significantly reduce the carbon footprint of AI technologies (12). Additionally, promoting the application of AI in environmental protection, resource management, and sustainable development—such as through smart pollution monitoring and optimized energy distribution—represents important pathways toward achieving greener AI. The construction industry plays a pivotal role in global energy consumption and carbon emissions, making the adoption of low-carbon building materials imperative for sustainable development. By advocating for these materials, the industry can significantly reduce its environmental impact. Low-carbon building materials offer a practical solution by decreasing building energy usage and greenhouse gas emissions. Through initiatives such as integrating renewable energy sources and utilizing efficient insulation materials, buildings can substantially decrease their reliance on fossil fuels, leading to a noticeable reduction in carbon emissions. This proactive approach addresses environmental concerns and sets the stage for a more sustainable and resilient built environment.

This study aims to explore public perceptions of the application of CNN models in the identification of low-carbon materials and how these perceptions influence the promotion and utilization of such materials. Although some research has addressed the application of AI technologies in environmental protection, studies focusing on public views of these innovative technologies and their impacts remain relatively scarce. Therefore, this research seeks to fill this gap, providing valuable insights for policymakers, businesses, and various sectors of society.

Public perceptions of these innovations are critical because they directly affect the acceptance and scope of technology application. Only when the public has a comprehensive understanding and trust in the accuracy and reliability of CNN models for low-carbon material identification can these technologies be applied in broader contexts. Furthermore, public attitudes directly influence the formulation and implementation of relevant policies, as well as corporate decisions regarding technology development and market promotion. In addition to environmental impacts, these technologies are closely related to public health. The use of low-carbon materials can reduce energy consumption and carbon emissions in buildings, thereby improving indoor air quality and decreasing the incidence of health issues such as respiratory diseases. Concurrently, the application of CNN models in medical image recognition has also achieved significant results, providing robust support for the early detection and treatment of diseases. Thus, exploring how these technologies intersect with public health not only enhances their relevance but also offers insights for broader application scenarios.

In light of the evolving landscape of smart cities, AI medical care, and low-carbon building materials, this study undertakes an exploration of their integration to bolster environmental sustainability within urban settings. The research endeavors to delve into the intersection of AI medical care and low-carbon building materials within smart city frameworks, aiming to fulfill several key objectives. Firstly, it seeks to conduct an in-depth analysis of the current applications and emerging trends of AI medical care and low-carbon building materials within the context of smart cities. Secondly, the study aims to scrutinize integration methods and mechanisms for seamlessly incorporating AI medical care and low-carbon building materials into the fabric of smart urban environments. Lastly, it endeavors to propose tangible strategies and actionable pathways for fostering synergy between AI medical care and low-carbon building materials within the context of smart cities. Through the execution of these objectives, the study introduces novel concepts and methodologies for smart city development and advocates for the wider adoption and advancement of AI medical care and low-carbon building materials. Thus, it makes significant strides toward the sustainable evolution of urban landscapes.

## 2 Literature review

The emergence of smart cities as a leading trend in global sustainable urban development underscores the profound impact of technological innovation on urban landscapes. As AI technology rapidly evolves, smart cities continually seek ways to harness its potential to optimize operations and enhance residents’ well-being. In tandem, the exploration and implementation of low-carbon building materials have become pivotal in shaping the infrastructural fabric of these forward-thinking cities (13). This section embarks on a comprehensive review of relevant literature spanning smart cities, AI medical care, and low-carbon building materials, laying the theoretical groundwork for the framework and objectives of this study. Smart cities epitomize a paradigm shift in urban development, leveraging cutting-edge information and communication technology alongside robust data analytics to efficiently manage resources and deliver essential services across various domains such as urban planning, transportation, environmental conservation, and energy management. Musa et al. (14) highlight key attributes including intelligent transportation systems, energy management, and environmental monitoring as integral components of smart cities. Ultimately, the overarching objective of smart city initiatives is to enhance operational efficiency, optimize resource utilization, and ultimately, improve the overall quality of life for residents. Through a synthesis of these interdisciplinary domains, this study aims to propel the advancement of smart cities toward greater sustainability and resilience in the face of urban challenges.

The integration of AI into the medical field has led to transformative breakthroughs. Research by Ghaffar Nia, Kaplanoglu (8) illustrates how AI empowers physicians by aiding in disease diagnosis, treatment planning, and predicting disease progression through the analysis of vast datasets and sophisticated machine learning algorithms. Moreover, Agarwal, Yadav (7) highlight the remarkable diagnostic accuracy achieved by AI-based medical care, particularly in areas like skin cancer detection and breast cancer screening, rivaling that of expert physicians. This rapid progression of AI in medical care holds immense promise for improving the precision and efficiency of medical diagnosis, reducing wastage of medical resources, and delivering superior medical care services to patients. As AI continues to evolve, its integration into medical practice stands to revolutionize patient care and enhance outcomes across diverse medical disciplines.

Low-carbon building materials play a pivotal role in mitigating greenhouse gas emissions and reducing resource consumption across their entire life cycle. Alaux et al. (15) underscore the importance of investigating and adopting such materials, emphasizing their potential to significantly decrease the construction industry’s energy dependency and mitigate carbon emissions from buildings. Building upon this, Norouzi et al. (16) highlight how the integration of low-carbon materials can effectively curb carbon emissions by lowering buildings’ energy consumption, especially through the incorporation of renewable energy sources and the utilization of high-efficiency insulation materials. The research and implementation effort not only contribute to the creation of energy-efficient and environmentally friendly urban environments but also drive sustainable urban development. By embracing low-carbon building materials, cities can reduce their ecological footprint and move toward a more sustainable future.

In regions such as the United States and Europe, the application of AI technology in the identification of low-carbon materials has also garnered widespread attention. These studies exhibit both similarities and differences in terms of algorithm optimization, dataset construction, and practical application scenarios when compared to research conducted in China. Comparative analysis reveals that research in different regions displays distinct characteristics and trends influenced by various factors, including cultural background, economic level, and policy environment. These differences provide a richer comparative perspective, facilitating a deeper understanding of the practical applications and potential challenges of AI technology in the identification of low-carbon materials.

In conclusion, the development of smart cities, AI medical care, and low-carbon building materials are pivotal considerations in contemporary urban sustainable development. The establishment of smart cities necessitates the incorporation of advanced technologies to elevate the management and service standards of urban areas. Meanwhile, the progression of AI medical care holds promise for enhancing the caliber and efficacy of medical services. Additionally, the adoption of low-carbon building materials emerges as a fundamental strategy for constructing energy-efficient and environmentally conscious cities. This study endeavors to investigate the integration of AI medical care and low-carbon building materials to bolster the environmental advantages of smart cities and contribute to the enduring sustainability of urban environments.

## 3 Methods

### 3.1 AI medical care in smart cities

The integration of AI into medical care within the smart city context heralds a new era of transformative benefits and possibilities. At its core, the synergy between AI and smart city infrastructure establishes a solid foundation for revolutionizing medical care delivery. The advanced intelligence and digitalization inherent in smart city infrastructure provide a fertile ground for AI applications in medical care. Medical care facilities within smart cities can capitalize on this robust technical framework to harness intelligent medical devices and systems, enabling seamless real-time collection, transmission, and analysis of medical data. This capability not only streamlines medical processes but also enhances the efficiency and effectiveness of medical care services. For instance, through continuous patient monitoring facilitated by intelligent devices, physicians gain unprecedented insights into patients’ physiological parameters, enabling remote diagnosis, prompt treatment adjustments, and ultimately, improved treatment outcomes. Furthermore, AI technology offers unparalleled advantages in medical imaging diagnosis, disease prediction, and diagnosis. Within the smart city ecosystem, medical care institutions can leverage sophisticated machine learning algorithms and big data analytics to glean invaluable insights from vast medical datasets. By doing so, they can refine the accuracy and precision of medical diagnoses to unprecedented levels. For example, AI-powered medical imaging diagnosis systems equipped with deep learning algorithms can autonomously analyze medical images, aiding physicians in rapid disease identification, minimizing diagnostic errors, and elevating diagnostic accuracy to new heights. Moreover, AI medical care in smart cities paves the way for personalized diagnosis and treatment strategies tailored to individual patient needs. By leveraging patients’ medical histories, genetic profiles, and lifestyle data, medical care institutions can harness AI algorithms to anticipate health risks and devise personalized preventive and treatment measures. This approach embodies the concept of precision medicine, wherein medical care interventions are finely tailored to each patient’s unique characteristics and circumstances (17, 18).

The integration of AI into medical care within the framework of smart city infrastructure represents a paradigm shift in medical care delivery, offering multifaceted benefits and transformative potential. At its core, the augmentation of intelligence and digitalization within smart city infrastructure establishes a robust technical environment conducive to the seamless collection, transmission, processing, and application of AI-driven medical services. This convergence unlocks a myriad of opportunities for intelligent, personalized, and precise medical care delivery, reshaping the landscape of medical care provision. Firstly, smart city infrastructure serves as a catalyst for seamless data collection and transmission, laying the groundwork for real-time monitoring and data acquisition. The heightened intelligence embedded in smart medical devices and sensors enables the continuous gathering of patients’ physiological parameters, condition data, and medical images. Leveraging the network infrastructure of smart cities, this wealth of data can be swiftly transmitted and shared, facilitating personalized medical services and furnishing invaluable insights for optimization and precision in medical care delivery. Secondly, the robust data processing and analysis capabilities afforded by smart city infrastructure empower medical care institutions to delve into vast medical datasets with unprecedented depth and efficiency. Through the utilization of information systems and big data platforms, medical care providers can harness formidable computing and storage capacities to process and analyze complex medical data. Cloud computing and big data technologies enable the exploration of patients’ health statuses and disease progression patterns, equipping physicians with enhanced diagnostic and treatment capabilities grounded in empirical evidence and scientific rigor. Furthermore, the intelligence and digitalization of smart city infrastructure enable the delivery of personalized medical services tailored to individual patient needs. By leveraging AI technologies and big data platforms, medical care facilities can scrutinize patients’ medical histories, genetic profiles, and lifestyle data to extract actionable insights. This facilitates the provision of tailored diagnosis and treatment plans, as well as personalized health management recommendations, thereby enhancing the precision and customization of medical services to unprecedented levels. In addition, smart city infrastructure supports the optimal allocation of medical resources, promoting the efficient utilization of medical care resources. By leveraging AI capabilities and big data analytics, medical institutions can analyze and forecast the supply and demand dynamics of medical resources. This enables the rational allocation of medical care resources, optimization of service layouts, and enhancement of service quality and efficiency, ultimately leading to improved patient outcomes and satisfaction. Lastly, the robust information security systems embedded within smart city infrastructure ensure the confidentiality and integrity of medical data. Privacy protection mechanisms safeguard against unauthorized access and misuse of medical information, preserving patients’ rights, interests, and privacy. This instills confidence among patients and medical care providers alike, fostering a secure environment for the exchange of sensitive medical information. In conclusion, the evolution of smart city infrastructure plays a pivotal role in advancing the integration of AI into medical care, ushering in a new era of intelligent and data-driven medical care delivery. By leveraging the capabilities of smart city infrastructure, medical care institutions can unlock unprecedented opportunities for personalized, precise, and efficient medical care provision, ultimately improving patient outcomes and shaping the future of medical care delivery (19–21).

The use of low-carbon building materials can significantly reduce carbon emissions during the operational phase of buildings, thereby improving urban air quality and decreasing health issues related to respiratory diseases caused by air pollution. Concurrently, the application of AI in healthcare, such as predicting disease risk through big data analysis and optimizing medical resource allocation, can further enhance public health and collectively promote the development of healthy cities alongside low-carbon materials. By collecting and analyzing vast amounts of environmental, health, and socioeconomic data, AI can construct complex models to predict changes in human health risks under various carbon emission scenarios. This analytical capability assists policymakers in accurately identifying high-risk areas and populations, enabling the formulation of effective emission reduction measures and health intervention strategies.

### 3.2 Application of low-carbon building materials in smart cities

Low-carbon building materials play a critical role in advancing sustainable construction practices, particularly within the context of smart cities. These materials encompass a diverse range of building elements meticulously engineered to minimize environmental impact and reduce greenhouse gas emissions throughout their entire life cycle, from production to disposal (22). With mounting concerns surrounding climate change, the adoption of low-carbon building materials has emerged as a pivotal trend within the construction industry. At its core, the adoption of such materials aligns with the overarching goal of decarbonization, seeking to address construction needs while mitigating the environmental footprint associated with building activities. This necessitates a multifaceted approach that encompasses various strategies, including the utilization of renewable resources, optimization of energy consumption, and reduction of emissions during manufacturing processes. For instance, substituting traditional building materials with recycled alternatives, implementing energy-efficient production techniques, and optimizing transportation methods for materials are all effective measures aimed at achieving low-carbon objectives (23). Table 1 (24) provides an overview of the key characteristics defining low-carbon building materials, illustrating their importance in fostering sustainable development within smart city environments.

**Table 1:** Concepts and characteristics of low-carbon building materials.

Characteristics	Description
Green and environmental friendly	Low-carbon building materials typically originate from renewable resources or possess recyclable properties, such as bamboo and recycled steel. They exhibit minimal environmental impact during production and use, thereby reducing the consumption of natural resources and mitigating environmental damage.
Energy efficiency	Low-carbon building materials demonstrate excellent energy utilization efficiency, thereby reducing energy consumption during the operational phase of buildings. For instance, thermal insulation materials with superior insulation properties contribute to the reduction of heating and cooling energy consumption in buildings.
Emission reduction	Low-carbon building materials prioritize the adoption of low-energy and low-emission production processes, often leveraging low-carbon fuels and energy sources. These practices effectively mitigate the release of harmful emissions, including greenhouse gasses, during the manufacturing process.
Recyclability	The design of low-carbon building materials emphasizes sustainability across the entire lifecycle, with a focus on material reuse and recycling. By facilitating material disassembly and recycling, these materials achieve recyclability, thereby reducing resource consumption and minimizing waste emissions.

The utilization of low-carbon building materials has profoundly influenced the urban environment and residents’ health in a multitude of ways, shaping a more sustainable and livable future for urban dwellers. Firstly, these materials play a pivotal role in improving air quality within urban areas. Crafted from eco-friendly components like natural fibers and recycled materials, low-carbon building materials minimize the emission of harmful substances, thereby ameliorating urban air quality. Compared to conventional building materials, these eco-friendly alternatives generate fewer pollutants during production and are more conducive to recycling post-use, alleviating environmental strain and reducing air pollution levels. Consequently, the health of urban inhabitants is enhanced as they breathe cleaner air, reducing the risk of respiratory illnesses and improving overall well-being. Secondly, the adoption of low-carbon building materials contributes to enhancing indoor environmental quality. These materials typically boast superior environmental adaptability and comfort attributes, such as effective thermal insulation, sound absorption, and moisture regulation capabilities. By improving indoor air quality and comfort, low-carbon building materials mitigate indoor air pollution and odors, creating healthier living environments for residents (25, 26). For instance, incorporating paints and construction materials with low volatile organic compound (VOC) levels diminishes the presence of hazardous substances in indoor air, reducing the risk of respiratory issues and enhancing overall health. Furthermore, the integration of low-carbon building materials aids in mitigating the urban heat island effect, a phenomenon where urban areas experience elevated temperatures compared to surrounding rural areas. Traditional materials like concrete and steel possess high heat capacity and thermal conductivity, exacerbating the urban heat island effect by absorbing and retaining solar energy. However, materials with low heat capacity and high reflectivity, such as reflective roofs and green roofs, effectively attenuate building and city temperature rises, ameliorating urban climates and bolstering residents’ living comfort. Additionally, the use of lightweight and recycled materials in construction reduces buildings’ structural weight, subsequently curbing their energy consumption and carbon emissions. By adhering to low-carbon building design principles, such as optimizing natural light utilization and promoting natural ventilation, buildings can further diminish their energy usage and dependence on external resources. This not only reduces greenhouse gas emissions but also promotes sustainable urban development by conserving energy and fostering resilience to climate change. Lastly, the integration of low-carbon building materials contributes to enhancing community health and residents’ well-being. By bolstering air quality, indoor environments, and urban climates, these materials mitigate the adverse impacts of environmental pollution and climate change on residents’ health, fostering community cohesion and enhancing overall quality of life. In essence, the utilization of low-carbon building materials represents a critical step toward creating healthier, more sustainable urban environments for present and future generations (27).

### 3.3 Integration of AI medical care and low-carbon building materials in smart cities

This study harnesses the power of AI and convolutional neural network (CNN) technology to revolutionize the prediction and design optimization of building materials. By constructing a sophisticated neural network model, the intricate correlations between building material performance and structure can be unraveled, drawing insights from extensive experimental datasets. This enables precise predictions of material performance under diverse design scenarios. For example, employing deep learning techniques facilitates the optimization of building material microstructures, ensuring the attainment of optimal mechanical properties and durability for enhanced structural integrity and longevity.

In the realm of deep learning, CNNs stand out as powerful tools for processing and analyzing grid-structured data tasks, particularly images and videos. Inspired by the human visual system, CNNs excel at extracting features from input data through layers of convolution and pooling operations, ultimately performing tasks such as classification or regression through fully connected layers. The relevance of CNNs to low-carbon building materials lies in their potential to revolutionize architectural design and material selection processes, paving the way for more environmentally friendly and sustainable construction practices. CNNs play a crucial role in analyzing the characteristics and performance of building materials. By inputting images of various materials into CNN models, their physical properties, structures, and textures can be classified and predicted with remarkable accuracy. This empowers designers and engineers to make informed decisions when selecting low-carbon building materials that meet specific requirements, thereby promoting sustainable building practices. Furthermore, CNNs contribute to optimizing the energy efficiency of buildings. By analyzing architectural appearance, layout, and material properties, CNNs can forecast building energy consumption and provide optimization recommendations. Adjusting window positioning and size, as well as modifying material thermal conductivity, based on CNN insights can significantly reduce energy consumption and minimize environmental impact. Moreover, CNNs are instrumental in the research and innovation of building materials. By analyzing existing materials’ characteristics and integrating extensive data and simulation experiments, CNNs aid scientists and engineers in developing novel low-carbon building materials. These materials may possess enhanced insulation properties, durability, and recyclability, thereby advancing the construction industry toward greater sustainability and eco-consciousness. In conclusion, CNNs offer immense potential in the exploration and implementation of low-carbon building materials. Leveraging CNN technology enables a deeper understanding and optimization of building material performance, enhances building energy efficiency, and propels the construction sector toward a more sustainable trajectory. This methodology aligns with global efforts to mitigate climate change and promote environmental conservation.

**Figure 1:**
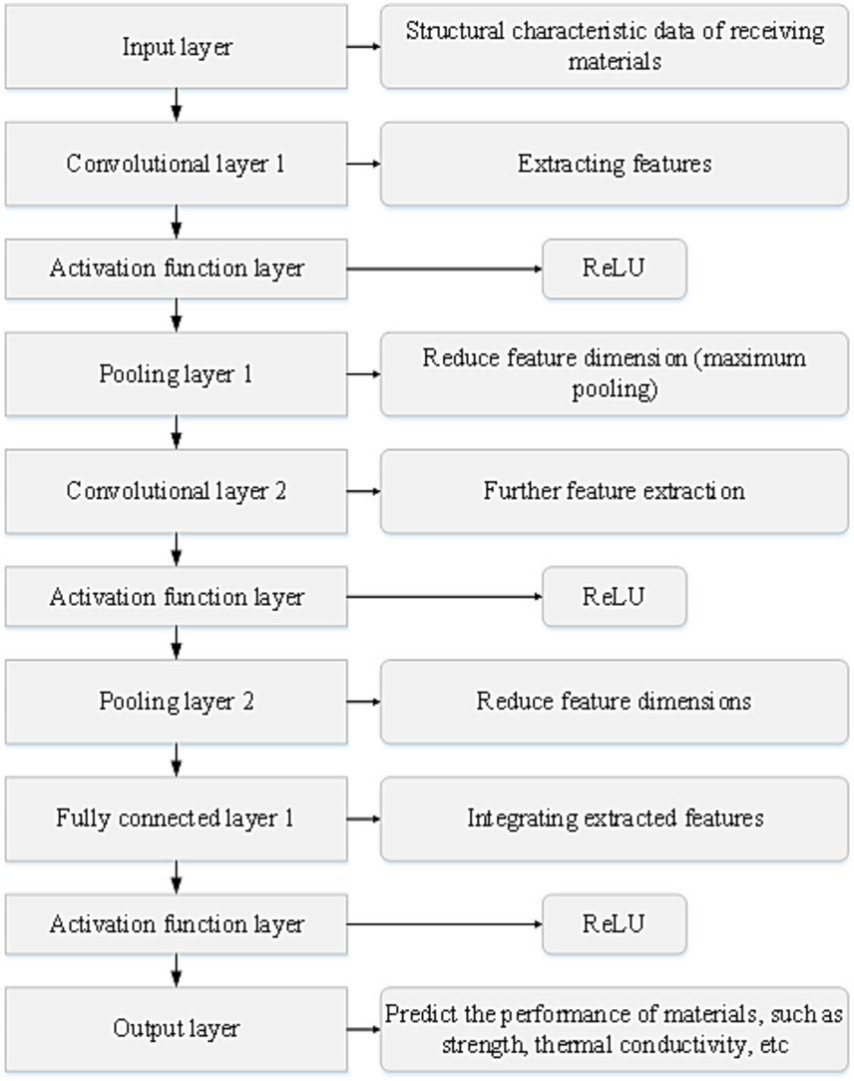
Process flow of the CNN model.

A CNN model is constructed to process the structural and performance data of materials, followed by obtaining the optimal design solution through model prediction and optimization. The model’s process flow is illustrated in Figure 1.

The specific steps are as follows:

1. Data collection and preprocessing: structural and performance data of building materials, including material composition, physical properties, mechanical performance, etc., were gathered. The collected data underwent preprocessing, including tasks such as data cleaning and normalization, to facilitate the training and optimization of the neural network.
2. Establishing the CNN model: the input of the neural network can consist of structural feature data of materials, such as crystal structure, element distribution, etc., while the output can be the predicted performance of materials, such as strength, thermal conductivity, etc. The model is constructed using convolutional layers, pooling layers, and fully connected layers. The output of the convolutional layer is calculated via Equation 1:

(1) $$z_{ij}^{l}=\overset{n^{l-1}}{\underset{m=1}{\sum }}\overset{f}{\underset{n=1}{\sum }}W_{mn}^{l}\;.\;x_{i+m-1,j+n-1}^{l-1}+b^{l}$$

In Equation 1, $z_{ij}^{l}$ represents the weighted sum of neurons in the i-th row and j-th column of the l-th layer, $W_{mn}^{l}$ is the weight of the convolution kernel, $x_{i+m-1,j+n-1}^{l-1}$ is the input data of the (l−1)*-*th layer, and $b^{l}$ signifies the bias term.

The Rectified Linear Unit (ReLU) activation function is defined as Equation 2:


(2) $$a_{ij}^{l}=\mathrm{ReLU}\left(z_{ij}^{l}\right)$$

In Equation 2, $a_{ij}^{l}$ represents the output of the neuron after the activation function, and ReLU denotes a commonly used activation function defined as $\mathrm{ReLU}\left(x\right)=\max\left(0,x\right)$.

The output of the pooling layer is calculated using Equation 3:


(3) $${\hat{x}}_{ij}^{l}=\mathrm{pool}\left(x_{ij}^{l}\right)$$

In Equation 3, ${\hat{x}}_{ij}^{l}$ represents the output of the pooling layer, and pool is the pooling function, such as Max Pooling or Average Pooling.

The output of the fully connected layer is calculated using Equations 4 and 5 below:


(4) $$z_{j}^{L}=\overset{n^{L-1}}{\underset{i=1}{\sum }}W_{ij}^{L}\;.\;a_{i}^{L-1}+b_{j}^{L}$$

(5) $$a_{j}^{L}=\mathrm{ReLU}\left(z_{j}^{L}\right)$$

Here, $z_{j}^{L}$ represents the weighted sum of the *j-th* neuron of the output layer, $W_{ij}^{L}$ refers to the weight connected to the output layer, $a_{j}^{L}$ signifies the output of the *j-th* neuron of the output layer, and $b_{j}^{L}$ denotes the bias term of the output layer.

1. Model training and optimization: training the neural network model involves utilizing collected data to learn the intricate relationship between material structure and performance. This process employs optimization algorithms, such as gradient descent algorithms, to refine model parameters. Through iterative adjustments, these algorithms minimize prediction errors and enhance the overall accuracy of the model, ensuring that it effectively captures the underlying patterns within the data.
2. Model verification and evaluation: following model training, an independent test dataset is employed to verify and evaluate the trained model. This step assesses the model’s generalization capability and prediction accuracy. Based on validation results, the model undergoes adjustments and enhancements to further improve its performance and reliability, ensuring its effectiveness in real-world applications.
3. Optimized design plan generation: the trained neural network model, now refined and validated, is deployed to predict and optimize building material structure and performance. By adjusting input parameters based on specific design requirements and objectives, the model generates an optimal design solution tailored to the project’s needs. This process ensures that the resulting design plan maximizes performance while adhering to predefined constraints and goals.
4. Plan verification and experimentation: the generated optimized design plan is translated into tangible building materials, and experimental verification ensues. Through rigorous testing and experimentation, the performance of the design plan is evaluated, and feedback from experimental outcomes is used to further refine and optimize the design. This iterative process establishes a closed-loop optimization cycle, continuously improving the design plan based on real-world feedback and ensuring its effectiveness in practice.

During the training of the CNN model, the following parameter settings are employed: the learning rate is set to 0.001 to control the model’s update speed during training; the batch size is set to 32; in each convolutional layer, the kernel size is 3 × 3; and the ReLU activation function is applied following the convolutional and fully connected layers to enhance the model’s nonlinear capacity.

For each type of building material, 30 samples are prepared, resulting in a total of 90 samples across three material types. Each sample includes 10 features, such as the composition ratio, manufacturing process parameters, and physical property indicators. The range for compressive strength testing is set at 20–100 MPa.

In the test set, the mean squared error (MSE) for the model’s compressive strength predictions is 2.5 MPa, while the MSE for flexural strength predictions is 1.2 MPa. In field tests, the error between the model’s predictions and the actual values falls within an acceptable range, indicating that the model exhibits strong predictive capabilities and practicality.

### 3.4 Questionnaire survey and parameter settings

#### 3.4.1 Questionnaire survey

This study utilizes a cross-sectional research design aimed at investigating public perceptions and attitudes toward smart cities, AI in healthcare, and low-carbon building materials at the current time point. Data are collected from 200 randomly selected respondents regarding their awareness, acceptance, and expectations in these areas. This design facilitates the acquisition of a snapshot of current public opinions, providing crucial insights into the image and status of these domains in the public mind. The survey is conducted from April 2022 to October 2022, spanning a duration of 6 months. Following the elimination of incomplete responses, 190 valid questionnaires are obtained, resulting in an impressive response rate of 95%. This high response rate ensures the reliability and validity of the survey findings. The questionnaire primarily includes sections on personal information, understanding and attitudes toward smart cities, knowledge and attitudes regarding AI in healthcare, attitudes and purchasing intentions toward low-carbon building materials, and satisfaction with proposed improvements to the survey materials. A random sampling method is employed to ensure that the sample adequately represents individuals of varying ages, genders, professions, and income levels. This approach aims to gather the broadest and most diverse public opinions possible.

In exploring perceptions of AI, a series of questions is designed to gain a deeper understanding of the public’s attitudes toward its applications in daily life. This includes inquiries about the perceived value of AI, concerns regarding potential risks, and views on its impact on employment. These questions aim to reveal the diverse perspectives held by the public regarding AI technology, ranging from enthusiastic support to reservations, and even concerns and opposition. For perceptions of smart cities, a set of questions is developed to assess the public’s understanding of the concept of smart cities and their views on the environmental impact of such initiatives. Special attention is given to identifying areas in which the public believes improvements are needed in the construction of smart cities, with the intent of guiding future policy development and practical implementation. Regarding views on low-carbon materials, the questions primarily focus on the public’s understanding of the concept and significance of low-carbon materials, their awareness of the environmental implications, and their willingness to choose low-carbon materials in everyday life, as well as the influencing factors. Additionally, the questionnaire includes several questions related to income level, such as annual household income and job stability, in order to indirectly assess the impact of economic status on perceptions and attitudes in these areas.

#### 3.4.2 Neural network parameter settings

In the training phase, a dataset comprising 1,000 sample data points is utilized, while an additional 200 sample data points are reserved for validation purposes. The learning rate employed during training is set at 0.01, ensuring a balanced approach to gradient descent optimization. The training cycle spanned 100 epochs, allowing the neural network model to iteratively learn and adapt to the underlying patterns in the data, ultimately enhancing its predictive capabilities and performance.

### 3.5 Ethic approval

The experiment was approved by Academic Ethics Review Committee of Guangzhou University (N^0^ GZRT2024169), China, on March 23, 2024. Our study did not involve animal or human clinical trials and was not unethical. In accordance with the ethical principles outlined in the Declaration of Helsinki, all participants provided informed consent before participating in the study. The anonymity and confidentiality of the participant guaranteed, and participation was completely voluntary. Participants volunteered to take part in the interview. Prior to participating in the interview, they were informed of the purpose of the study and were told that “submission of records” was considered informed consent. Participants could withdraw at any time during the participation process.

## 4 Results

### 4.1 Descriptive statistics

#### 4.1.1 Personal information of the survey subjects

**Figure 2:**
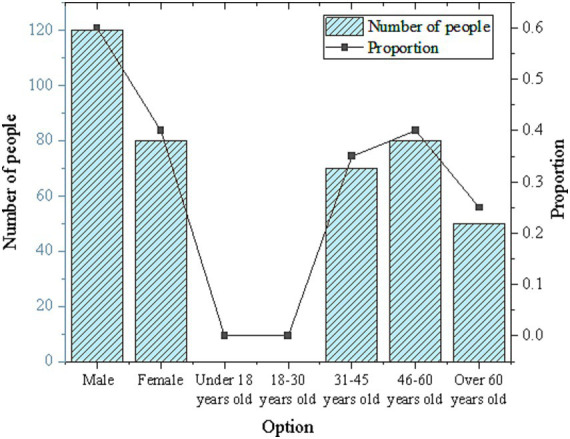
Personal information statistics results.

The demographic data collected from the survey participants is summarized in Figure 2.

As depicted in Figure 2, males constitute 60% of the survey respondents, while females make up 40%. The age distribution is predominantly concentrated between 31 and 60 years old. These findings indicate a relatively balanced gender ratio among participants, with the majority falling within the middle-aged demographic. This demographic profile enables a more nuanced understanding of attitudes and perspectives regarding smart cities, AI medical care, and low-carbon building materials across different demographic groups.

#### 4.1.2 Smart city statistics

**Figure 3:**
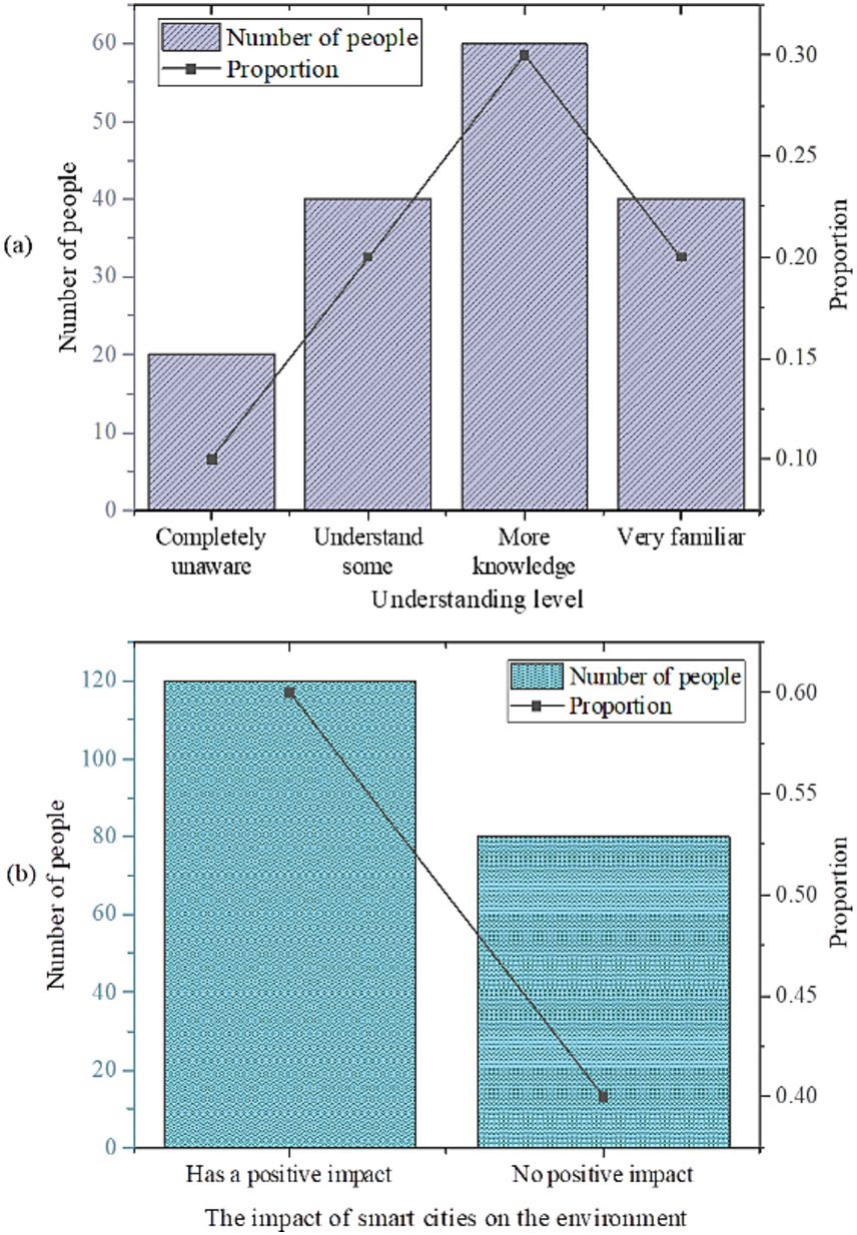
Descriptive statistical analysis results of the questionnaire. **(a)** Understanding level; **(b)** The impact of smart cities on the environment.

The statistical findings regarding smart cities are presented in Figure 3.

As illustrated in Figure 3, 80% of the respondents possess an understanding of smart cities, with 30% indicating a high level of comprehension. Additionally, 60% of the participants perceive smart cities to have a positive impact on the environment. These results suggest that a considerable majority of the respondents exhibit a certain level of familiarity with smart city concepts, and a notable portion acknowledges their beneficial influence on environmental conservation and sustainable living. This trend likely signifies an increasing awareness of sustainable development and environmental stewardship, as well as a favorable disposition toward the advancement of smart city initiatives. Such insights hold significant implications for guiding future urban planning and developmental endeavors.

#### 4.1.3 AI medical statistics

**Figure 4:**
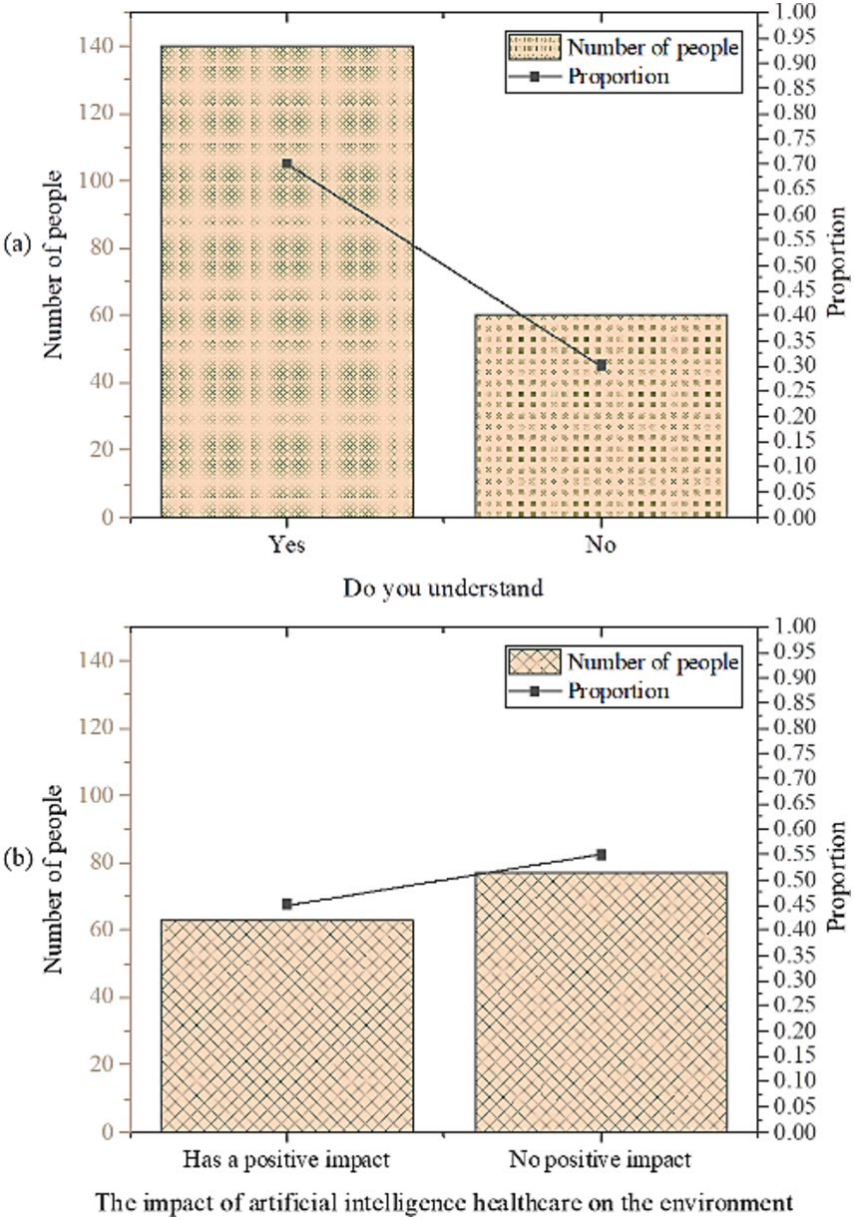
Statistical results of AI medical treatment [**(a)** understanding of AI medical care; **(b)** impact of AI medical care on the environment].

The statistical findings regarding AI medical treatment are presented in Figure 4.

As depicted in Figure 4, 70% of the respondents exhibit an understanding of the application of AI in the medical field, with 45% expressing belief in its positive impact. Through optimizing supply chain management, enhancing energy efficiency, and promoting the rational allocation of medical resources, AI contributes to reducing the negative environmental impact of healthcare. Proponents also emphasize that AI can drive innovation in medical technology, leading to the development of more environmentally friendly and efficient medical devices and treatment methods. These innovations not only improve the quality of healthcare services but also mitigate environmental damage. This indicates a substantial level of comprehension among participants regarding the integration of AI in medical care, with a notable portion acknowledging its potential environmental benefits. However, 30% of respondents remain unfamiliar with this domain, and 55% perceive its impact as unfavorable, possibly reflecting apprehensions and uncertainties surrounding the adoption of novel technologies. Some skeptics argue that while AI can enhance the efficiency of healthcare services, the computational resources and energy consumption required for its operation are substantial. If these resources are not managed and utilized effectively, they may pose adverse environmental impacts. This skepticism primarily stems from several factors: first, the public’s limited understanding of AI technology results in insufficient awareness of its potential environmental implications; second, there is significant concern regarding privacy and security issues in the healthcare sector, with fears that the introduction of AI technology may introduce new risks; third, varying levels of acceptance of low-carbon and environmentally friendly concepts among the public lead to differences in expectations and attitudes toward the application of AI in healthcare. These findings underscore the necessity for enhanced dissemination and advocacy of AI in medical care. Moreover, it underscores the importance of gauging public perception and attitudes toward its prospective environmental implications.

#### 4.1.4 Statistics of low-carbon building materials

**Figure 5:**
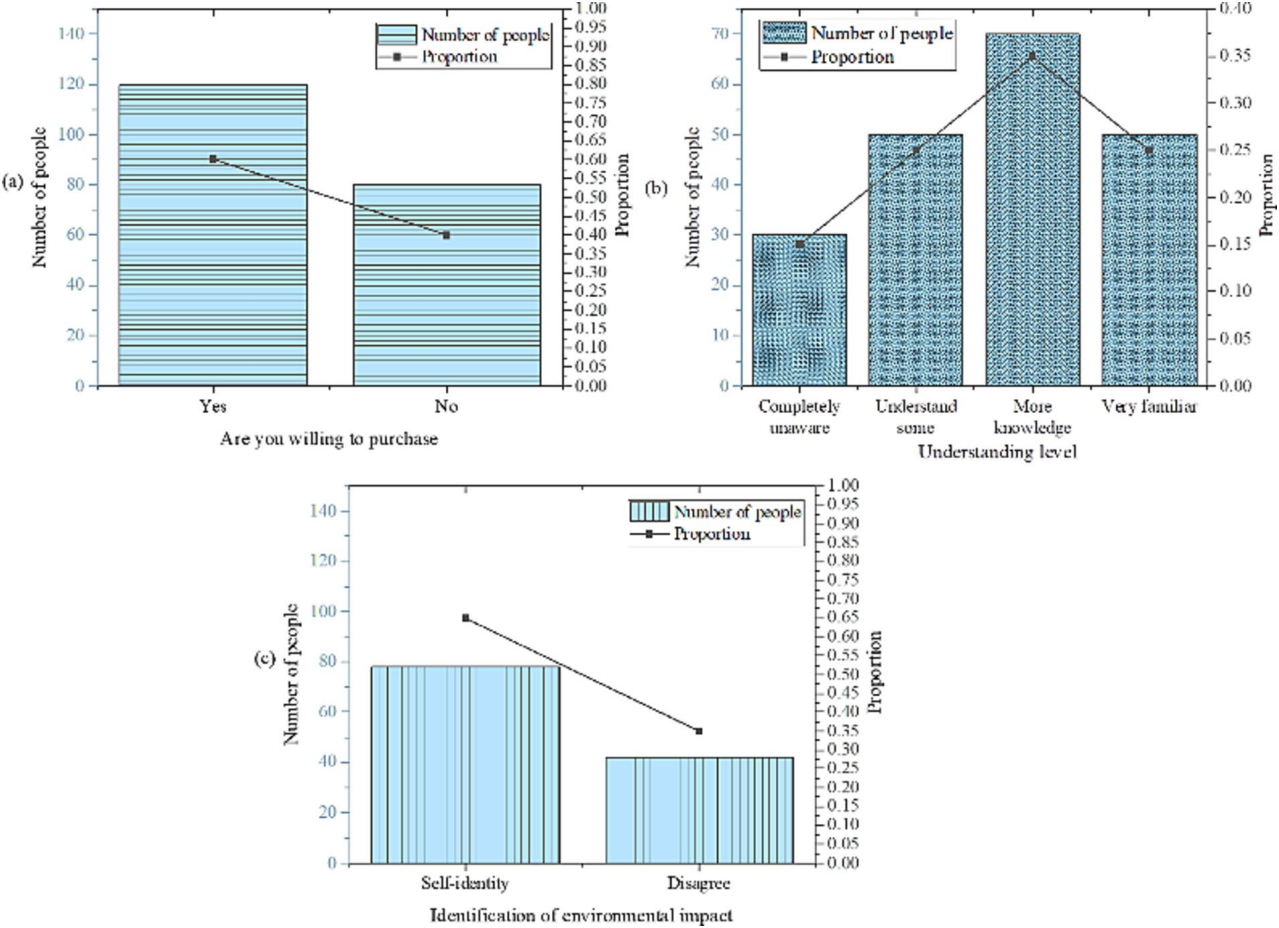
Statistical results of low-carbon building materials [**(a)** willingness to purchase; **(b)** degree of understanding; **(c)** recognition of environmental impact].

The statistical analysis of low-carbon building materials is illustrated in Figure 5.

As depicted in Figure 5, 60% of respondents have expressed their willingness to pay higher prices for purchasing low-carbon building materials, indicating a notable level of recognition of this material type. Furthermore, 30% of respondents possess a substantial understanding or extensive knowledge regarding low-carbon building materials. However, 35% of respondents exhibit limited knowledge, signifying the necessity for further publicity and dissemination efforts. Regarding their recognition of environmental impact, 65% of respondents concur that low-carbon building materials can have a positive influence. These findings suggest that while there exists a certain level of awareness regarding low-carbon building materials, concerted efforts to enhance public awareness and underscore their environmental benefits are imperative to bolster widespread acceptance and recognition of their adoption.

#### 4.1.5 Satisfaction survey

**Figure 6:**
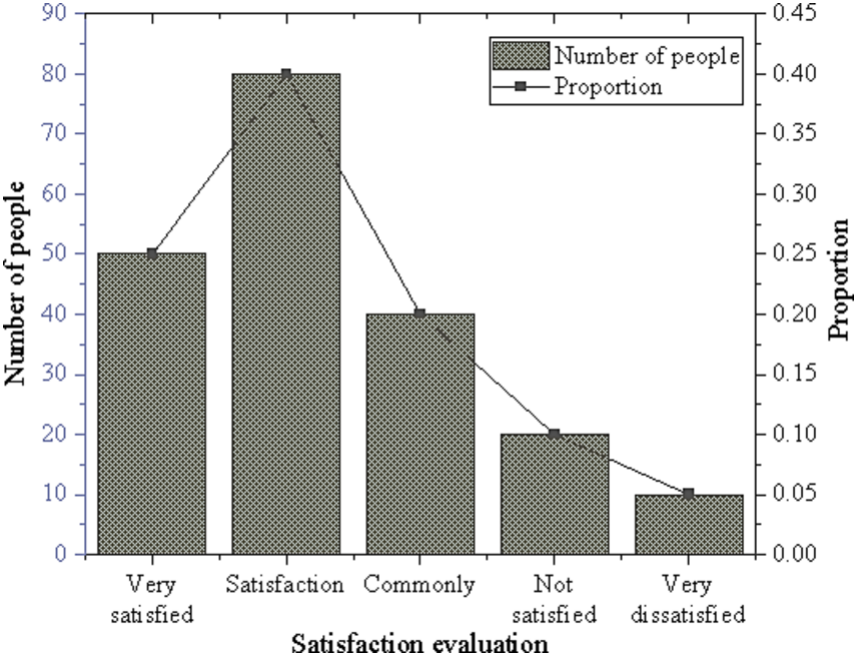
Satisfaction survey results on material improvements.

The satisfaction level regarding material improvements was analyzed, and the results are presented in Figure 6.

As depicted in Figure 6, 40% of participants expressed satisfaction, while 25% reported being very satisfied. Additionally, 20% exhibited a neutral attitude, while the proportions of dissatisfaction and extreme dissatisfaction were 10 and 5%, respectively. Overall, a majority of participants expressed satisfaction or high satisfaction levels with the survey. This indicates a notable level of recognition for the survey content, as well as the information or services provided throughout the survey process.

### 4.2 Neural network model performance analysis

**Figure 7:**
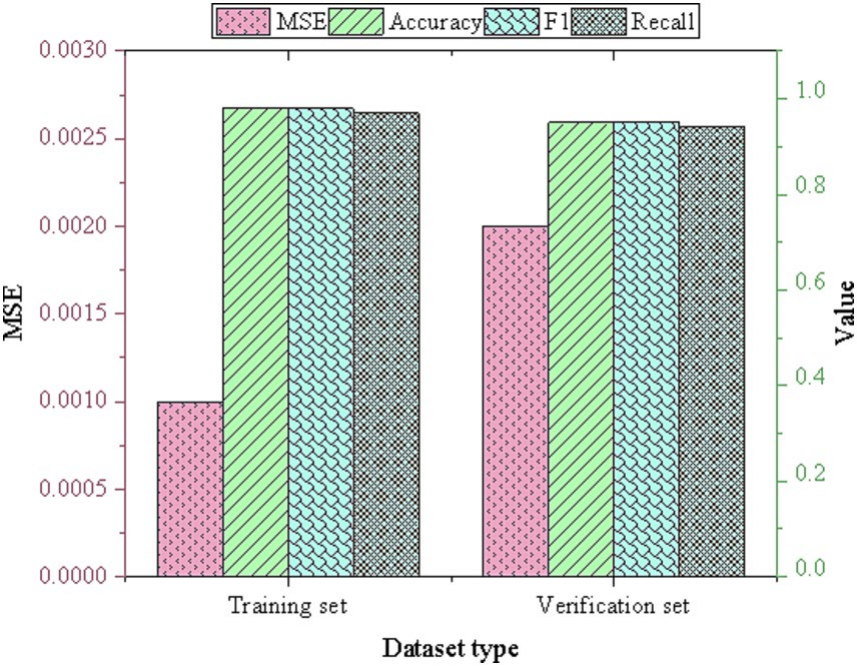
Neural network model performance results.

The CNN model constructed was trained and verified, yielding the following results, as depicted in Figure 7.

As depicted in Figure 7, the model showcases exceptional performance on the training set, boasting remarkably high precision, recall, and F1 scores. Despite a slight decline in performance observed on the validation set, the model exhibits commendable generalization capabilities, with accuracy and recall rates consistently exceeding 95%. Notably, the MSE of the model remains remarkably low, indicating minimal disparity between predicted and actual values. Specifically, the MSE on the training set is a mere 0.001, indicative of a robust fit to the training data. Even with a slightly higher MSE observed on the validation set (0.002), the model maintains strong performance, highlighting its capacity to predict new data with precision and reliability. This result is consistent with the findings of Guan and Wang (28). These results underscore the effectiveness and reliability of the model in handling unseen data and generalizing well beyond the training set.

## 5 Discussion

In cultures that emphasize collectivism and obedience to authority, individuals may be more inclined to accept decisions made by AI, whereas cultures that prioritize individualism and free will may exhibit greater vigilance regarding the potential threats that AI poses to individual rights. Environmental awareness varies across different cultural contexts. In cultures with a strong emphasis on environmental protection, individuals may be more concerned about the potential impacts of new technologies, such as AI, on the environment and are more likely to support technological applications that contribute to environmental conservation. In Europe, the application of AI places a significant focus on privacy protection and ethical standards, underscoring the importance of sustainable development and social responsibility in AI technology. Germany has made remarkable progress in intelligent manufacturing and Industry 4.0 while actively promoting the application of AI in fields such as healthcare and education. In Asia, particularly in China, Japan, and South Korea, there has been rapid development in the application of AI technologies. These countries have achieved significant breakthroughs in areas such as smart cities, smart homes, and smart finance, particularly benefiting from clear advantages in big data processing, cloud computing, and Internet of Things technologies. Denmark is recognized as one of the leading countries in global wind energy production, achieving a green transformation of its energy structure and low-carbon development through the large-scale utilization of renewable energy sources like wind. Denmark’s wind energy projects serve as successful models for the global advancement of low-carbon energy.

In the field of intelligent transportation, the application of AI significantly enhances the efficiency and safety of urban traffic systems. By integrating big data analytics and machine learning algorithms, AI can predict traffic flow in real time, optimize traffic signal control, reduce congestion, and assist in planning more efficient public transportation routes. Furthermore, the continuous development of autonomous driving technology, which relies on AI decision-making systems, is expected to greatly improve road safety and decrease traffic accidents. When combined with IoT technologies, AI can enable intelligent connectivity between vehicles and infrastructure, further enhancing the overall effectiveness of transportation systems.

In energy management, the application of AI contributes to the development of greener and more efficient energy systems within smart cities. Utilizing deep learning and other technologies, AI can forecast energy demand and optimize energy distribution, thereby improving energy utilization efficiency and reducing waste. Additionally, in conjunction with smart grid technology, AI can monitor and control electricity supply in real time, ensuring the stable operation of the grid and facilitating rapid energy dispatch when necessary. In the renewable energy sector, AI can also optimize the utilization of clean energy sources, such as solar and wind power, promoting the optimization and upgrading of urban energy structures. Regarding smart city governance, the application of AI offers novel perspectives and tools for urban management. Through big data analysis and natural language processing techniques, AI can collect and analyze urban operational data in real time, providing scientific support for government decision-making. AI can assist in monitoring urban environmental quality, predicting disaster risks, and optimizing the allocation of public service resources.

The return on investment for smart cities and low-carbon materials may be extended, necessitating collaborative efforts and long-term commitments from government, enterprises, and individuals. As the advancement of intelligence and low-carbon initiatives progresses, employment opportunities in traditional industries may decline, while new job openings in emerging sectors could increase. This transition may lead to instability in employment structures and social stratification. The implementation of smart cities and low-carbon materials relies on advanced technologies and information systems, which could result in an over-reliance on technology, potentially impacting the autonomy and diversity of social life. The perspectives of policymakers are crucial in the execution of proposed strategies. Policy support and guidance can stimulate the enthusiasm of businesses and individuals, facilitating the widespread adoption of smart cities and low-carbon materials. Regulatory frameworks and oversight are essential for maintaining market order and ensuring the legal and compliant use of technology. During the implementation of smart cities and low-carbon materials, construction companies must focus on cost control and benefit assessment to ensure the economic feasibility and sustainability of projects. Although the production costs of low-carbon materials may exceed those of traditional materials, the energy savings and emissions reduction benefits, along with long-term economic returns during their use, must also be considered. Buildings constructed with low-carbon materials exhibit superior performance in insulation, thermal regulation, and lighting, leading to reduced energy consumption and operational costs. Governments should formulate both long-term and short-term strategic plans that clearly delineate the roles and significance of AI and low-carbon materials in economic development, environmental protection, and social progress. These strategic plans should encompass various aspects such as technology research and development, industry cultivation, policy support, and talent training to ensure coherence and systemic alignment in policy formulation.

In the field of healthcare, the application of AI often relies on extensive personal health data. However, numerous ethical challenges arise during the collection, storage, and processing of this data, particularly concerning data privacy. Personal health data is highly sensitive, and its breach can pose severe privacy risks to individuals. Malicious actors may exploit this data for fraud, identity theft, or other illicit activities. Even in cases where data is not directly disclosed to external entities, inadequate management and protection can lead to misuse within organizations. Healthcare institutions or researchers might access or utilize this data without authorization for unethical research or commercial purposes.

The training data for AI systems often originates from specific groups or environments, which may introduce bias when algorithms process data from different groups or contexts. If the training data predominantly comprises individuals of a particular race or gender, the algorithm may exhibit biases when handling data from other races or genders. Participants may tend to overestimate their understanding of smart cities, AI, and low-carbon materials. This overestimation may stem from various factors, such as social desirability bias (where respondents feel pressured to display a higher level of understanding of emerging technologies), memory biases (where respondents may struggle to accurately recall or assess their knowledge), or cognitive biases (where respondents may hold inflated self-evaluations of their abilities or knowledge). To address this, it is essential to clearly communicate the study’s objectives and significance to respondents before data collection, as well as the potential biases in self-reported data. By increasing respondents’ awareness, they can be encouraged to more authentically reflect their knowledge levels, thereby minimizing instances of overestimation or underestimation.

The performance of CNN models may be influenced by factors such as dataset quality, training methodologies, and parameter settings. As the model has not been tested in real-world environments, its performance in practical applications remains uncertain. To mitigate the impact of these limitations, methodologies such as cross-validation, model tuning, and performance evaluation are employed during the research process to enhance the model’s accuracy and generalization capabilities.

## 6 Conclusion

This study employs a multifaceted approach, combining both quantitative and computational methods, to delve into perceptions and attitudes toward key aspects of urban development: smart cities, AI medical care, and low-carbon building materials. Through a combination of questionnaire surveys and neural network modeling, this study endeavors to provide a comprehensive understanding of public sentiments and expectations in these domains. This study employs a random sampling method to ensure the diversity and representativeness of the sample. Efforts are made to include respondents from various age groups, genders, occupations, and regions to minimize the impact of self-selection bias. The survey findings reveal a generally positive outlook toward smart cities and low-carbon building materials among participants, highlighting an increasing awareness and acceptance of sustainable urban development practices. However, there exists a nuanced understanding and some skepticism regarding AI medical care, suggesting a need for further education and awareness campaigns in this area. AI is expected to facilitate the widespread adoption of personalized medical services by analyzing information related to patients’ genetics, lifestyle habits, and medical histories to provide tailored treatment plans and health management recommendations. This approach is anticipated to significantly enhance the efficiency and effectiveness of healthcare services while reducing medical costs. Strengthening collaboration among academia, industry, and healthcare institutions is essential for advancing the research and application of AI technologies. By sharing resources, experiences, and knowledge, the innovation and dissemination of technology can be accelerated.

The findings of this study indicate that participants generally held a positive attitude, suggesting an increasing awareness and acceptance of sustainable urban development practices among the public. This conclusion underscores the significant role of smart cities in promoting urban sustainability and provides robust support for policymakers to further advance the construction and development of smart cities. In the realm of AI in healthcare, although the public possesses a certain level of understanding regarding its applications, there are nuanced perceptions and some skepticism. This reflects concerns regarding the potential risks and uncertainties associated with new technologies. Therefore, it is imperative to enhance educational and promotional activities in the future to improve public comprehension of AI in healthcare and alleviate concerns. The CNN model developed here demonstrates robust performance, exhibiting high prediction accuracy on both the training and validation sets. Despite its strengths, limitations such as a relatively small sample size and scope of survey questions, as well as the lack of verification on a larger scale, may impact the representativeness and reliability of the results. Future research endeavors could focus on expanding the sample size, refining survey question formulation, validating the model’s generalization capabilities on a larger scale, and conducting more comprehensive real-world analyses and evaluations of smart city initiatives, AI medical care, and low-carbon building materials to provide actionable insights for public health and sustainable urban development. Future research will expand the sample size to encompass a broader population and geographical areas. This endeavor will facilitate a more comprehensive understanding of public perceptions and attitudes toward these domains, thereby enhancing the representativeness and applicability of the research findings.

## Data Availability

The raw data supporting the conclusions of this article will be made available by the authors, without undue reservation.
